# Palladium-103 plaque radiation therapy for retinal angioma

**DOI:** 10.1016/j.ajoc.2025.102482

**Published:** 2025-11-20

**Authors:** Paul T. Finger, Andrew Lukban, Ren-Dih Sheu, Robert D. Stewart, Julie R. Bloom, Kenneth E. Rosenzweig

**Affiliations:** a The Department of Ocular Tumor, Orbital Disease and Ophthalmic Radiation Therapy, The New York Eye Cancer Center, New York City, New York, USA; b The Departments of Ophthalmology and Radiation Oncology, Mount Sinai Hospital, Icahn School of Medicine at Mount Sinai, Department of Ocular Tumor and Orbital Disease, USA; c The Department of Ophthalmology, Division of Ocular Oncology, Tulane University School of Medicine, USA

**Keywords:** Retina, Angioma, Palladium-103, Plaque, Radiation

## Abstract

**Purpose:**

Plaque radiation has been used in the treatment of angiomas of the choroid, retina, and vasoproliferative tumors of the retina. Herein, is presented the novel use of palladium-103 (^103^Pd) plaque radiation as primary treatment for retinal angioma.

**Observation:**

Two patients presented with solitary peripheral retinal angiomas. Both initially progressed on observation and failed subsequent argon laser photocoagulation. Pre-treatment comparative dosimetry was performed to evaluate the available radiation sources palladium-103 (^103^Pd), iodine-125 (^125^I), and yttrium-90 (^90^Y) for an equivalent tumor apex dose). Yttrium-90 plaque therapy offered the most favorable, measured intraocular dose distribution to the fovea, optic disc and lens for both patients. However, informed consent led to a patient preference for ^103^Pd, due to its prior and more long-term follow-up after irradiation of choroidal hemangiomas, melanoma, and retinoblastoma. Each was treated to ^103^Pd plaque apical doses of 50 and 60 Gray (Gy) over a 7-day period. Doses to fovea were very low at 1.3 and 1.7 Gy respectively. Both angiomas regressed over 1-year observation as evidenced by decreased tumor size, with resolution of retinal exudates, overlying retinal hemorrhage, and a localized retinal detachment. Vision was unchanged.

**Conclusion and importance:**

^103^Pd plaque brachytherapy was utilized to treat two peripheral retinal vascular angiomas. Calculated doses to fovea and optic disc suggest no risk of radiation maculopathy or optic neuropathy. ^103^Pd plaque brachytherapy was found to control tumor growth and reverse their exudation resulting in preservation of vision.

## 1 Introduction

Retinal angiomas are a benign process that may occur spontaneously or in association with von Hippel-Lindau (VHL) disease.^1^ Patients with a family history of VHL who are younger or exhibit multifocal retinal angiomas should undergo genetic VHL testing as well as an initial and subsequent periodic systemic survey for non-ocular vascular and/or malignant tumors.^1^ In contrast, patients with spontaneous retinal angiomas may have systemic hypertension, anemia, sickle-cell retinopathy, or peripheral retinal vascular diseases. Despite their origin, the ophthalmic goals of retinal angioma management are to preserve vision and the eye.^1^, ^2^, ^3^

Small inactive retinal angiomas, without sight-threatening exudates or retinal detachment, can be safely observed.^4^ However, eye tumor specialists have employed laser, cryotherapy, radiation, and intraocular surgery to treat actively growing, exudative lesions, with or without a secondary retinal detachment.^1^^,^^5^, ^6^, ^7^, ^8^, ^9^, ^10^, ^11^, ^12^, ^13^, ^14^ The decision to use one form of treatment versus another is typically based on tumor size, location, and tumor-exudation-related risks to vision.

Treatment decisions are made in consideration of how quickly each modality is likely to result in tumor regression, vision preservation, and stabilization of the eye. For example, laser often takes multiple monthly sessions and is more difficult to perform through exudate and retinal detachment or when the tumor is juxtapapillary.^5^, ^6^, ^7^, ^8^, ^9^ Cryotherapy takes one or more sessions, but each treatment harbors a risk of acute secondary exacerbation of exudates and retinal detachment prior to regression.^9^

Ruthenium-106 (^106^Ru) plaque radiation takes days, is surgical, and has been employed for solitary retinal angiomas with and without anti-vascular endothelial growth factor or vitreoretinal surgery.^11^, ^12^, ^13^, ^14^ External beam radiation techniques are available but the stationary beam is affected by eye movements (mobile target volume) and must travel through the anterior segment, adnexa and orbit to reach the tumor.^15.16^

In a literature review on October 22nd, 2025, utilizing the keywords retinal angioma, palladium-103 (^103^Pd), iodine-125 (^125^I), ruthenium-106 (^106^Ru), and yttrium-90 (^90^Y) plaque, radiation therapy; we could find no prior reported cases of retinal angiomas treated with ^103^Pd, ^125^I, or ^90^Y. Herein, we present our initial experience treating two unifocal spontaneous exudative retinal angiomas utilizing ^103^Pd plaque brachytherapy.

### 1.1 Case presentations

#### 1.1.1 Case #1

A 41-year-old female was symptomatic of a “shadow” in the upper left visual field of the left eye for several weeks’ duration. Her past medical history was significant for systemic hypertension and anemia due to hemorrhagic fibroids. She was referred for evaluation of a rounded white retinal tumor in her left eye, and there was no history of retina capillary hemangioma or VHL disease.

**Fig. 1:**
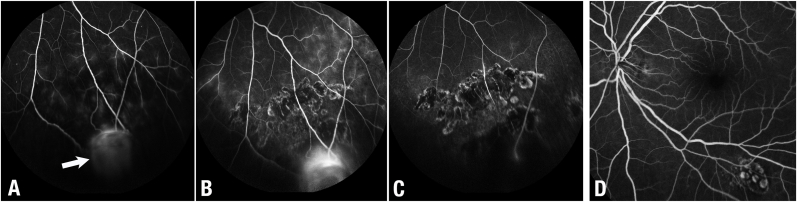
**Case 1:** Fluorescein angiographic images over time. A, prior to laser the retinal capillary angioma (white arrow) was inferior to a localized area of capillary nonperfusion, frosted retinal vessels and scattered microaneurysms. B, the next image depicts the tumor after a combination of scatter and demarcation argon laser. Note nasal and temporal extension of the peritumoral disease. C, One-year after palladium-103 plaque fluorescein angiography reveals relative hypoperfusion of the tumor, reductions in peritumoral retinal leakage and frosted retinal vessels. Even some of the larger retinal vessels have closed after irradiation. D, the slightly separated image reveals the macula at 1-year. Note the exudates have cleared.

At presentation visual acuities were 20/20 in the right eye and 20/16 in the left. The anterior segments were normal, with symmetrical intraocular pressures (IOPs) at 18 mm/Hg in both eyes, with normal slit lamp biomicroscopy without vasculopathy. Posterior segment examination revealed mild hypertensive changes in both eyes. However, in the infero-temporal quadrant of the left eye, there was a tumor surrounded by exudate and a very small retinal detachment. Ultrasound imaging revealed a dome-shaped tumor with variable internal reflectivity, a small retinal detachment and no extrascleral tumor extension. The tumor was measured to be 5.2 × 4.1 mm in transverse and longitudinal basal diameter, and 2.3 mm in height. Fluorescein angiography revealed a hyperfluorescent tumor surrounded by a poorly perfused peripheral retina, scattered microaneurysms, and spots of focal hyperfluorescence (Fig. 1). Sector panretinal photocoagulation and demarcation argon laser was applied around the tumor. Both uveitis and genetic screening testing were negative.

**Fig. 2:**
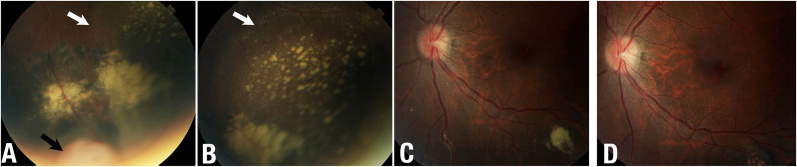
**Case 1:** A, B, C, the 3 fundus photographs on the left demonstrate the condition of the tumor (black arrow) and retina just prior to palladium-103 plaque brachytherapy. Despite laser treatment, there were increased exudates and subretinal fluid (white arrows) around the tumor. Exudate had progressed posterior to the equator. D, the slightly separated photograph was taken 1-year after palladium-103 plaque radiation therapy. Note the resolution of the perivascular and focal posterior segment exudates.

Three months after presentation, examination revealed no significant change in visual acuity or IOP. The tumor minimally increased in apical height to 2.6 mm. At 6 months though her visual acuity, IOPs, and anterior segment examinations were stable, there now was a massive increase in exudation and retinal detachment superotemporal to the tumor (Fig. 2). Ultrasonography revealed an increased tumor height to 2.9 mm, and a larger base diameter at 5.6 x 4.7 mm. Documented growth and progression of exudate led to a discussion of the relative risks and potential benefits of observation, additional laser photocoagulation, cryotherapy, and irradiation (various source plaque and proton).^15^, ^16^, ^17^, ^18^, ^19^ At The New York Eye Cancer Center and related hospitals, available plaque sources included ^103^Pd, ^125^I, and ^90^Y. In accordance with the American Brachytherapy Society Guidelines, a pre-operative comparative plaque dosimetry was performed to aid in the determination of which isotope would be the most favorable for each patient (Table 1).^20^ This comparison revealed that the use of ^90^Y would allow for the lowest dose of radiation to the fovea and optic disc. However,^103^Pd afforded less radiation to critical intraocular structures compared to ^125^I. In addition, the use of either ^103^Pd or ^125^I would allot for larger plaque sizes, and thus larger tumor-free safety margins as compared to ^90^Y.^21^ Lastly, our patient was informed of the relative lengths of follow up and outcomes after treatment.

**Table 1:** Comparative plaque dosimetry.

Isotope	Case #1				Case #2			
	Distance	^125^I	^103^Pd	^90^Y	Distance	^125^I	^103^Pd	^90^Y
Location		Dose in Gray (Gy)				Dose in Gray (Gy)		
Lens Center	8.3	19.3	17.3	0.3	10.4	12.8	10.5	0.0
Optic Disc	22.0	2.4	1.2	0.0	17.3	3.1	1.7	0.0
Fovea	21.4	2.6	1.3	0.0	17.6	3.1	1.7	0.0
Opposite Retina	23.0	2.1	1.1	0.0	23.0	2.2	1.1	0.0
Inner Sclera	1.0	110.1	113.1	234.8	1.0	111.8	114.1	323.5
Axial 5 mm Point	6.0	26.9	24.7	3.0	6.0	25.5	27.3	4.1
Tumor Apex	2.9	60.0	60.0	60.0	3.6	50.0	50.0	50.0

^103^Pd = palladium-103, ^125^I = iodine-125, ^90^Y = yttrium-90, Doses in Gy (Gray).

For comparison, High-Dose-Rate (HDR) ^90^Y doses are displayed in Low-Dose-Rate (LDR) equivalent doses 17, 18.

Intraoperatively, scleral transillumination was used to place episcleral markings at the tumor base and 2–3 mm safety margin. Then a 12-mm Collaborative Ocular Melanoma study gold plaque containing ^103^Pd seeds was affixed to the eye wall covering the targeted zone. Intraoperative ultrasound imaging was used to confirm proper plaque placement. The patients received continuous radiation over 7 days, after which the radioactive plaque was surgically removed. The tumor was 21.4 mm from the fovea and 22 mm from the optic disc, resulting in 1.3 Gy to the fovea, 1.2 Gy to the optic disc, 17.3 Gy to the lens center, 1.1 Gy to the opposite retina, and 113.1 Gy to the sclera; tumor apex received 60 Gy (Table 1). These collateral doses to fovea and optic nerve did not cause radiation maculopathy or optic neuropathy.

Follow up examinations revealed stable visions and IOPs in the irradiated eye. Both subretinal fluid and exudates demonstrated progressive improvement at 2, 4, and 6 months after ^103^Pd plaque (Fig. 1, Fig. 2). Specifically, the exudative retinal detachment resolved at 4 months and the exudates by 1-year. Tumor thickness decreased to 2.4 mm at 4 months, and 2.1 mm at 1 year. The patient's last visual acuity was stable at 20/16.

#### 1.1.2 Case #2

A 72-year-old male with hypertension and diabetes, has a past ocular history of cataract surgery, macular pucker, and pars plana vitrectomy in the right eye. He noted an allergy to fluorescein and no family history of cancer. He was referred for an asymptomatic vascular tumor in his right eye. His systemic and ophthalmic workup did not uncover an etiology.

**Fig. 3:**
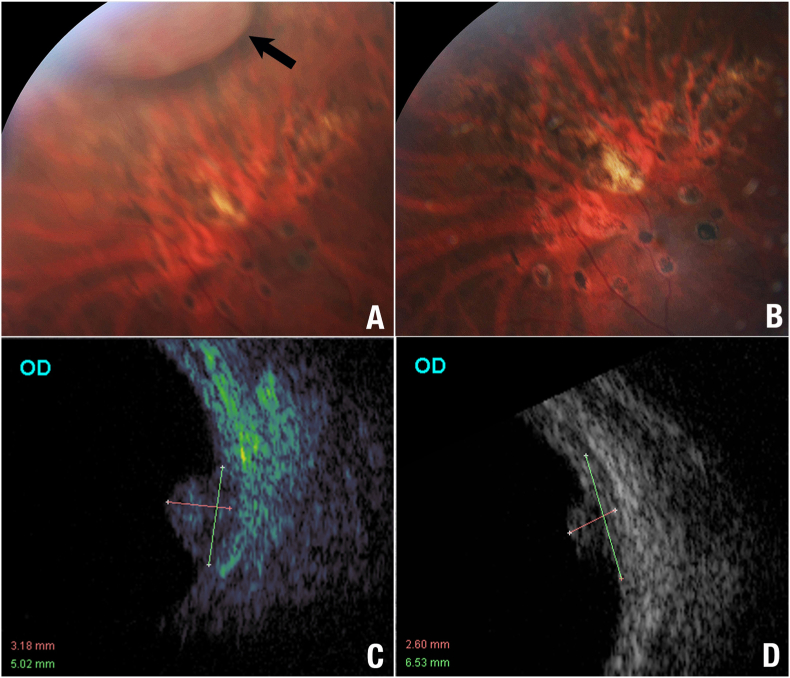
**Case 2:** Fundus photographs, A, note the retinal capillary hemangioma (black arrow) anterior to laser demarcation and prior to palladium-103 plaque therapy. B, the top right image reveals the same section 1-year after palladium-103 plaque brachytherapy. The tumor has regressed. C, an ultrasonographic B-scan image shows the retinal capillary hemangioma prior to irradiation. D, a B-scan shows the regressed tumor. The adjacent choroid thickening is likely due to intraretinal exudate or edema.

He had a macular epiretinal membrane and a visual acuity of 20/32. In the supero-temporal mid periphery, there was a vascular tumor, with gross overlying hemorrhage and trace exudate and surrounding subretinal fluid (Fig. 3). Ultrasound imaging revealed a dome shaped, variably reflective, tumor with a thickness (to inner sclera) of 3.2 mm and a base of 5.0 x 3.1 mm. He returned to his referring doctor for circumferential laser, missed a return appointment at The New York Eye Cancer Center, and represented 8 months later. His vision, IOP, and anterior segment examinations were stable. However, while the posterior segment examination revealed laser demarcation and decreased tumor hemorrhage, ultrasound imaging showed an increased tumor height to 3.2 mm, and base of 5.0 × 5.3 mm, there was no retinal detachment (Fig. 3).

Three months later, the tumor appeared darker, and ultrasonography revealed documented growth in both tumor height and base (3.6 mm height, base 5.3 × 5.5 mm). The potential risks and benefits of continued observation, cryotherapy, and radiation were discussed. We noted that potential plaque sources included ^103^Pd, ^125^I, ^90^Y as well as comparative dosimetry and relative, historical lengths of patient follow-up who chose ^03^Pd. Using the same plaque surgical technique as described for Case #1, this patient's tumor received an apex dose of 50 Gy; his macula received 1.7 Gy, disc 1.7 Gy, lens 10.5 Gy, opposite retina 1.1 Gy, and sclera 114.1 Gy. These low radiation doses delivered to the vision critical structures were related to the distance of the tumor to the fovea (17.6 mm) and optic disc (17.3 mm), respectively and did not cause radiation maculopathy or optic neuropathy. At 6 months follow up, his tumor had regressed to 3.0 mm height. At 1 year there were no exudates or retinal detachment, the tumor had regressed to 2.6 mm, and his vision was stable at 20/32.

## 2 Discussion

Small progressive retinal angiomas are typically treated with multi-session laser photocoagulation to the posterior feeder vessel(s) followed by direct applications as needed. Recalcitrant, poorly visible, and larger retinal angiomas have been managed with double freeze-thaw cryotherapy or vitreoretinal surgery. Plaque radiation therapy has been employed alone or in combination with vitreoretinal surgery.

### 2.1 Comparison of cryotherapy and plaque radiation therapy

Cryotherapy is a single surgical procedure performed under ophthalmoscopic visualization. This technique uses rapid freezing to create intracellular ice formation, leading to vascular stasis and cell lysis. The process also involves the extracellular ice drawing water out of the cells, causing them to shrink and collapse, which releases cytotoxic proteins and chemicals. As the ice melts, water reenters the cells, causing them to burst. This results in acute secondary vasculitis which exacerbates the exudation of intravascular blood, serum, and fats.^9^^,^^22^ Vision loss associated with cryotherapy often results from this acutely increased exudation and hemorrhage, which may lead to secondary retinal detachment and traction.

In contrast, widely available low-dose-rate plaque radiation therapy (^103^Pd, ^125^I, ^106^Ru), typically administered over 5–7 days, requires two surgical procedures—one for inserting the plaque and another for its removal.^20^ Radiation produces hydroxyl radicals and induces DNA damage, triggering apoptosis and vascular sclerosis over time.^23^^,^^24^ Unlike cryotherapy, the immediate effects of plaque radiation on intraocular tumors are relatively mild. However, there exist intermediate and long-term dose-dependent effects which include DNA damage to vascular endothelial and pericyte cells, resulting in vascular leakage, closure, subacute inflammation, and thrombosis. Late effects include vascular intimal fibrosis, capillary dropout, ischemia, hypoxia, atrophy, and tumor necrosis.^23^^,^^24^ Both cryotherapy and plaque irradiation have proved effective in treatment of progressive and exudative retinal angiomas.^25^ However, this study highlights the importance of individualized treatment approaches tailored to specific tumor characteristics and intraocular location.

Retinal angiomas were treated to apical ^103^Pd plaque radiation doses of 60 and 50 Gy, respectively (Table 1). While posterior segment complications were not expected at the calculated ^103^Pd doses, doses to lens are likely to promote cataract formation (Table 1). Though early, at one year's follow-up there have been no visually significant radiation side effects. This study suggests that ^103^Pd plaque radiation therapy can be used to treat retinal angiomas.

## CRediT authorship contribution statement

**Paul T. Finger:** Writing – review & editing, Writing – original draft, Validation, Resources, Project administration, Methodology, Investigation, Funding acquisition, Formal analysis, Data curation, Conceptualization. **Andrew Lukban:** Writing – review & editing, Methodology, Investigation, Data curation. **Ren-Dih Sheu:** Writing – review & editing, Investigation, Formal analysis, Data curation. **Robert D. Stewart:** Writing – review & editing, Methodology, Investigation, Formal analysis, Conceptualization. **Julie R. Bloom:** Writing – review & editing, Validation, Investigation, Formal analysis. **Kenneth E. Rosenzweig:** Writing – review & editing, Validation, Resources, Methodology, Formal analysis.

## Patient consent

Both patients consented to publication of the case in writing. This report does not contain any personal information that could lead to the identification of the patient. Therefore, the case conforms to the Tenet's of Declaration of Helsinki and the Health Insurance Privacy and Portability Act. It has been approved for publication by The New York Eye Cancer Center's IRB and Ethics committee.

## Intellectual property

We confirm that we have given due consideration to the protection of intellectual property associated with this work and that there are no impediments to publication, including the timing of publication with respect to intellectual property. In doing so we confirm that we followed the regulations of our institutions concerning intellectual property.

## Research ethics

We further confirm that any aspects of the work covered in this manuscript that has involved human patients has been conducted with the ethical approval of all relevant bodies and that such approvals are acknowledged within the manuscript.

## Authorship

All authors attest that they meet the current ICMJE criteria for authorship.

## Funding

This research was supported by The Eye Cancer Foundation http://eyecancercure.com.

## Declaration of competing interest

The authors declare the following financial interests/personal relationships which may be considered as potential competing interests:Dr. Finger owns patents and controlling stock interest in a company that competes with companies that make palladium-103 seeds. However, the manuscript clearly states why the patients chose palladium-103 versus the source produced by his company, and offers a positive outcome using his competitors medical device.
